# Maintenance of Genetic Diversity of Black Sea Bream despite Unmonitored and Large-Scale Hatchery Releases

**DOI:** 10.3390/biology11040554

**Published:** 2022-04-02

**Authors:** Te-Hua Hsu, Hung-Tai Lee, Hsueh-Jung Lu, Cheng-Hsin Liao, Hong-Yi Gong, Chang-Wen Huang

**Affiliations:** 1 Department of Aquaculture, National Taiwan Ocean University, Keelung 20224, Taiwan; realgigi@mail.ntou.edu.tw (T.-H.H.); hygong@mail.ntou.edu.tw (H.-Y.G.); 2 Center of Excellence for the Oceans, National Taiwan Ocean University, Keelung 20224, Taiwan; 3 Department of Environmental Biology and Fisheries Science, National Taiwan Ocean University, Keelung 20224, Taiwan; hungtailee@gmail.com (H.-T.L.); hjlu@mail.ntou.edu.tw (H.-J.L.); chliao@mail.ntou.edu.tw (C.-H.L.)

**Keywords:** microsatellites, genetic diversity, genetic effect, aquaculture, Sparidae

## Abstract

**Simple Summary:**

Stock enhancement aggressively replenishes depleted wild finfish populations. However, stock enhancement of black sea bream in Taiwan with complex genetic sources, especially when successful, maintains genetic diversity but dramatically changes the genetic structure within and among wild populations.

**Abstract:**

Stock enhancement, used for replenishing depleted wild finfish populations, is an aggressive approach. Stock enhancement projects in Taiwan involve black sea bream (*Acanthopagrus schlegelii*), a major commercial species. During 2004–2015, even management agencies conducted stock enhancement projects, leading to numerous private releases that have not been recorded. Stock enhancement by a private hatchery without accurate genetic records may lead to a genetic structure change in wild populations. Using allele frequencies at nine microsatellite loci, we studied the genetic effects of stock enhancement in 19 samples collected from populations in the hatcheries and the wild. In 458 individuals from nine hatchery samples, most populations showed weak but significant genetic differences and complex clusters in structure analysis, indicating dramatic stock change within and among hatcheries. The 10 wild populations (*n* = 773) also had a complex genetic composition and were genetically different among sampling sites and times. However, a simple and clear cluster in structure analysis was found for only one sampling site, which had no release history. Thus, stock enhancement with complex genetic sources helps maintain genetic diversity but dramatically changes the genetic structure within and among wild populations, especially when stock enhancement is successful.

## 1. Introduction

Advances in fishing technology have led to fish stocks, which are renewable fishery resources, being exhausted. Approximately half of all fish stocks have been deemed “fully exploited” or “overexploited” [1,2]. Pollution and habitat destruction due to human activities have drastically reduced the abundance and distribution of marine fish and invertebrate populations [2,3,4] and depleted fishery stocks in more than 100 species to date [5,6,7].

Aiming to improve fishery resources, Taiwan’s government promotes a massive stock enhancement program in its coastal waters every year [8,9]. Under this program, numerous artificial breeding fry are released, with a small number of fish labeled to enable survival rate evaluation [8,9,10]. Moreover, the government annually allocates a considerable amount of money to this program. However, the role of genetic factors in stock enhancement has been neglected [6,11,12]. Conventional markers (biological, physical, and chemical) cannot estimate the reproduction rate of released fish [5,6,13]. Stock enhancement programs should incorporate genetic information such as genetic stock structure and diversity. The official fishery organization (the Taiwan Fisheries Sustainable Development Association; TFSDA) in Taiwan procures fish fry for stock enhancement from one or several private hatcheries. According to several genetic studies, negative effects on natural populations were noted after successful stock enhancements [7,14]. These enhancements involved the release of hatchery populations or the escape of numerous relatively unfit [14,15,16,17] and not genetically diverse individuals, such as the Adriatic sturgeon [18], Korean starry flounder [19], and black sea bream from Japan [20]. The genetic effects of these hatchery fish in the wild have received considerable attention in developed countries. For example, Japan, the United States, and several countries in Europe have created official agencies for managing stock enhancement [7,21,22]. These agencies not only verify the reliability of external markers, but also evaluate the genetic composition of offspring and their reproductive rate through mark–recapture studies [5,7,22]. However, identifying fish sources is difficult when release records are complex and unclear, such as in Taiwan [8,9,10,11,12].

Black sea bream (*Acanthopagrus schlegelii*), an economically vital species for both fisheries and aquaculture, is widely distributed along West Pacific coasts from Japan and Korea to the East China Sea and Taiwan. In southern China, black sea bream males become sexually mature within 1 year, and 50% of them change sex by two years old. In this fish, reproduction occurs at 1–2 years of age in coastal waters and at river mouths [23]. The species is abundant off the west coast of Taiwan, where it is a popular sport fish. Notably, concern over the rapid decline in black sea bream stocks is growing because the related catch production declined from 718 tons in 2000 to 212 tons in 2015, according to the Fisheries Statistical Yearbook of Taiwan [24]. Black sea bream aquaculture began in the 1980s, and Taiwanese hatcheries are mainly located in the Kaohsiung and Ping-tung areas. Broodstocks are from main fishery areas, which are off the west coast of Taiwan (Chiayi–Yunlin–Changhua). Using reliable mass production techniques [8,9], massive hatchery juveniles are released into the wild to replenish the insufficient natural supply [8,9,10]. Black sea bream has thus become a dominant species for stock enhancement in Taiwan. During 2004–2015, more than 12 million hatchery black sea bream fry were released into the coastal water off Taiwan by TFSDA [8,9,10,11,12].

Although no genetic or hatchery information is available for stocks and fry in Taiwan during 2004–2015, investigating the genetic structure of hatchery and wild populations and assessing the effectiveness of stock enhancement are critical [8,9,10,11,12]. The analysis data allow study of the genetic diversity of fish fry and tracing of fish origins in the absence of hatchery information [11,12]. In this study, we used microsatellite DNA markers to distinguish cultured black sea bream populations from wild ones and to understand the genetic effects of stock enhancement on these wild populations.

## 2. Materials and Methods

### 2.1. Sample Preparation

**Figure 1:**
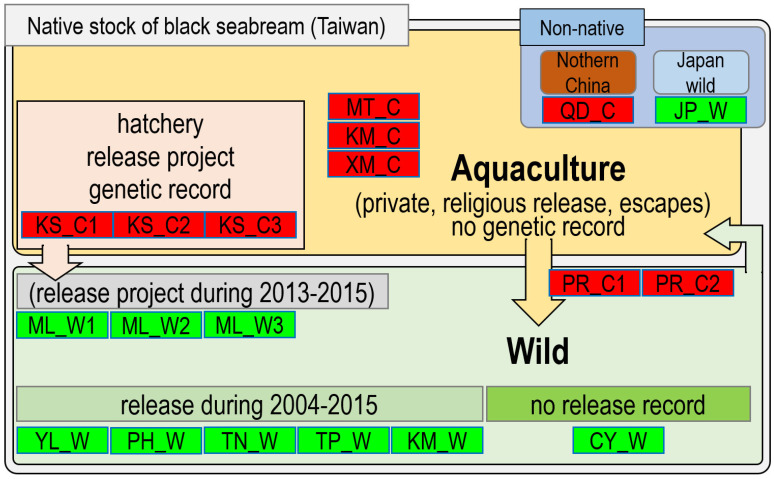
Sampling, hatchery, and stock enhancement information for black sea bream.

**Figure 2:**
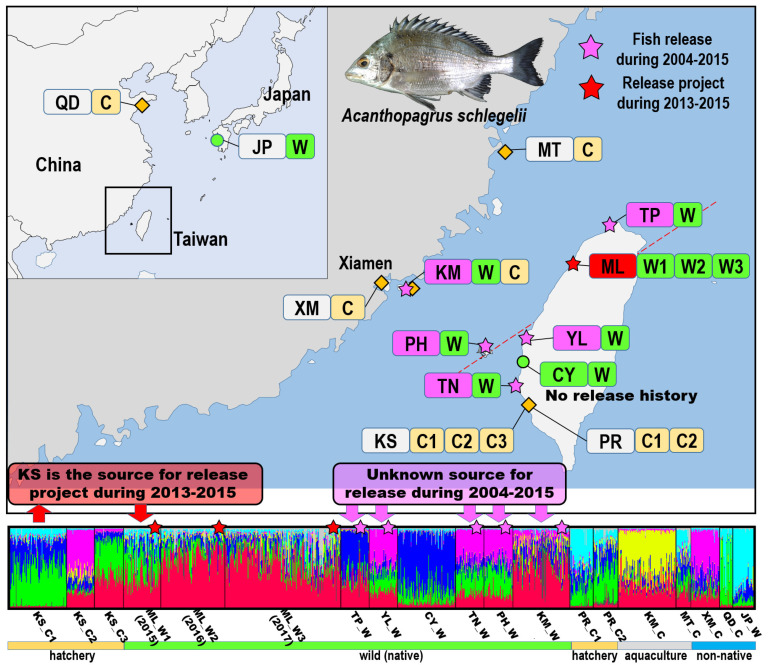
Sampling locations and structure analysis of 19 black sea bream populations. KS: Kaohsiung City, private hatchery which provides juveniles for official release; PR: Kaohsiung City, from unknown hatchery for private release; TP: Taipei city; ML: Miaoli County; YL: Yunlin County; CY: Chiayi County; PH: Penghu County; TN: Tainan City; KM: Kinmen County; MT: Matsu islands; XM: Xiamen city; QD: Qingdao city; and JP: Nagasaki Prefecture. The estimated population structure based on the highest probability structure run at K = 8. Each individual is represented by a thin vertical line, which is partitioned into K colored segments that represent individuals’ estimated likelihood of membership in each of the K clusters.

In total, 1231 black sea bream specimens (458 from the hatcheries and 773 from the wild) were obtained from 2015 to 2017 (Table 1). Species were identified by following the method of Hsu et al. [25]. Fresh specimens—at least 20 individuals for each batch, including broodstock, juveniles, and subadults/adults—were sampled from three types of hatchery source: (1) a private hatchery for the TFSDA release project and without genetic information (KS_C1, KS_C2, and KS_C3); (2) an unknown hatchery for private (religious) release (PR_C1 and PR_C2); (3) aquaculture farms from offshore islands of Taiwan (KM_C and MT_C) and southern China (XM_C); and (4) an aquaculture farm from northern China (QD_C). Ten batches from eight field locations: (1) Miaoli County, site of the TFSDA release project during 2013–2015 (ML_W1, ML_W2, and ML_W3); (2) Yunlin County, Penghu County, Tainan City, and Taipei City, with a hatchery fish release history during 2004–2015 (YL_W, PH_W, TN_W, TP_W, and KM_W); (3) Chiayi County, with no hatchery fish release history during 2004–2015 (CY_W); and (4) Nagasaki Prefecture, Japan, used for comparison (JP_W; Table 1; Figure 1). The geographical locations of these populations, sampling locations with abbreviated population names, and sample size for each population are presented in Figure 1 and Figure 2 and Table 1. Small muscle tissue pieces (approximately 3–5 mm) were prepared from fresh (2% alcohol used for anesthesia) or frozen fish specimens, transported to our laboratory for molecular study, and preserved in 95% ethanol. The standard proteinase K/phenol method modified from an animal DNA extraction protocol was used. Moreover, DNA template quality was assessed through 0.8% agarose gel electrophoresis.

### 2.2. SSR Markers

In accordance with Hsu et al. [26], nine microsatellite markers for black sea bream were selected and validated through multiplex polymerase chain reaction (PCR) to determine those ideal for genetic analysis (Table 2). PCR amplification was performed in 20 µL reaction volumes containing 5–10 ng of template DNA, 1× PCR buffer (10 mM Tris and 50 mM KCl, pH 9.0), 200 μM of each dNTP, 1.5 mM MgCl_2_, 0.5 U of Taq polymerase (Promega, Madison, WI, USA), and 4 pmol of each primer. Thereafter, PCR cycling was performed in an Autorisierter Thermocycler (Eppendorf, Hamburg, Germany) with initial denaturation at 95 °C for 2 min, which was followed by 30 cycles of denaturation at 95 °C for 30 s, annealing at locus-specific temperatures for 30 s, extension at 72 °C for 30 s, and a final extension at 72 °C for 10 min. The fragments in PCR products were analyzed using an ABI 3130 Genetic Analyzer (Applied Biosystems, Foster City, CA, USA), with the output analyzed using GeneMapper software (version 4.0, Applied Biosystems, Foster City, CA, USA).

### 2.3. Population Genetic Analysis

The observed genetic diversity (*Ho*), expected genetic diversity (*He*), and *F_IS_* were calculated using Genalex 6.41 [32]. The chi-square Hardy–Weinberg equilibrium (HWE) test results, pairwise *F_ST_* values, and associated *p* values were also determined using Genalex 6.41 [32]. To determine *F_ST_* significance levels, multilocus genotypes were randomized between sample pairs (9999 permutations), and the significance after Bonferroni correction was calculated [33,34]. Genotyping errors and null alleles were estimated using Microchecker 2.2.3 [35]. To elucidate a population’s genetic structure on the basis of multilocus genotypes, an admixture model with correlated allele frequencies was developed using Structure version 2.3.4 [36,37]. Five independent runs were performed for the entire data set for K values (numbers of groups) ranging from 1 to 9. All runs were based on 1,000,000 iterations of burn-in, followed by an additional 5,000,000 iterations. The best estimation of the K value was obtained using Structure Harvester [38]. Summation and graphical representation of the structure analysis results were performed using Clumpak [39] and Structure Plot version 2.0 [40], respectively. The population structure estimation was based on the highest-probability structure obtained at K = 8. Hierarchical analysis of molecular variance (hierarchical AMOVA) was performed to partition the total genetic variance within and between regions, as described by Excoffier et al. [41]. Arlequin was used to perform AMOVA. The most-supported grouping can be automatically detected using SAMOVA (based on Arlequin 3.5) [42,43]. We calculated pairwise relatedness (unrelated, half-sibling, full-sibling, or parent–offspring) between each pair of individuals by using RELATED [44] and Wang’s [45] estimator. The expected relatedness values (r) were 0.5 between full-sibling pairs and parent–offspring pairs, 0.25 between half-sibling pairs, and 0 between unrelated individuals [44]. We prepared a kinship network by using a pairwise relatedness (r) of >0.4, which indicates related individuals [46]. Parentage assignment between the 94 broodstock (KS_C1) and 192 wild (recaptured) samples (ML_W3) was estimated using Cervus 3.0.7 [47]. For accuracy, parentage was assigned by employing a minimum of nine loci, 0.9; proportion of loci typed, 0.01; proportion of loci mistyped, 0.01; error rate in likelihood calculations, 0.01; and simulation of 100,000 offspring at an 80% confidence interval applying the LOD confidence determined.

## 3. Results

### 3.1. Genetic Diversity within Populations

**Figure 3:**
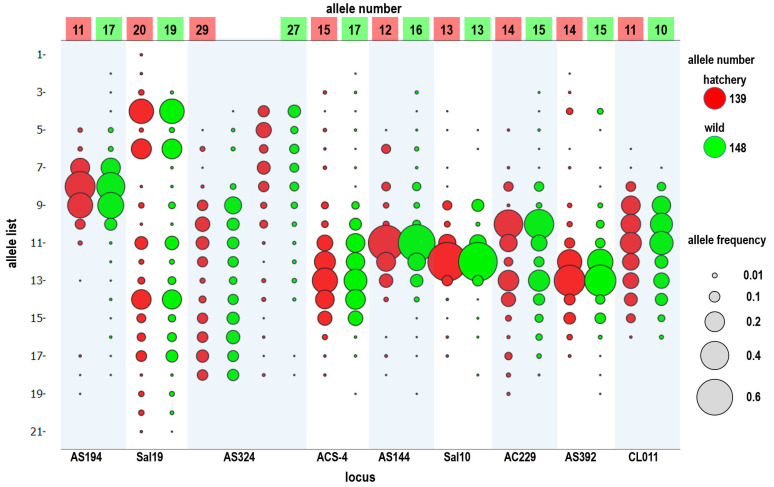
Allele frequency of nine microsatellite loci in hatchery (*n* = 388) and wild (*n* = 739) black sea bream populations in Taiwan. Hatchery: KS_C1, KS_C2, KS_C3, PR_C1, PR_C2, KM_C, and MT_C; Wild: ML_W1, ML_W2, ML_W3, YL_W, PH_W, TN_W, TP_W, KM_W, and CY_W.

Across the nine microsatellite markers, all the populations were successfully genotyped. No monomorphic loci were found among the 19 populations. In total, 170 alleles were detected from the samples (*n* = 1231). In all individuals, the marker AS324 exhibited the highest number of alleles per locus (31 alleles), whereas the marker CL011 exhibited the lowest number (12 alleles; Table 3). Fewer alleles were found in the hatchery populations (*n* = 388; 139 alleles) than in the wild populations (*n* = 739; 148 alleles); however, the allele frequency pattern was similar (Figure 3).

The marker AS324 in PH_W exhibited the highest number of alleles per locus and highest allele richness (21 alleles and 13.921, respectively), whereas the marker SaI10 in KM_C exhibited the lowest (4 alleles and 1.387, respectively; Table 4 and Table 5). The average heterozygosity (*Ho*) over all loci was between 0.565 and 0.725 among the 19 populations. CY_W exhibited the highest expected heterozygosity for all the loci, whereas XM_C exhibited the lowest (Table 4 and Table 5). The mean estimates of expected heterozygosity (*He*) over all loci were between 0.683 and 0.742 among the 19 populations. PR_C2 exhibited the highest expected heterozygosity for all the loci and XM_C and KS_C3 the lowest (Table 4 and Table 5). Of the 171 population–locus combinations, 67 displayed deviations from the *HWE* significant at the *p* < 0.001 level (Table 4 and Table 5), with no strong trends of deviation observed for specific loci (between 5 and 10). No possible genotyping errors were noted in any loci, and null alleles were found to be possible for only one locus (AC229) in KS_C1 (Table 4 and Table 5). The average *F*_IS_ was between −0.051 and 0.164 among the 19 populations. The lowest *F*_IS_ was found in TP_W and the highest in XM_C (Table 4 and Table 5).

### 3.2. Genetic Differentiation among Populations

Pairwise comparisons between sampling locations were performed. The pairwise *F_ST_* values ranged from 0 (KS_C1–ML_W1) to 0.056 (XM_C–JP_W), and most of them were significant (*p* = 0), suggesting small but significant genetic differentiation (Table 6). Most comparisons among the 19 populations indicated low genetic differentiation (below 0.04), but the results of comparisons of other locations with QD_C and JP_W were high (QD_C–others: 0.012–0.052; JP_W–others: 0.018–0.056) (Table 6). Pairwise comparisons among Taiwan hatchery populations obtained values ranging from 0.005 to 0.037 (average 0.023); among Taiwan wild populations, 0.005 to 0.034 (average 0.017); and among Taiwan hatchery–wild populations, 0 to 0.039 (average 0.020; Table 6).

### 3.3. Genetic Structure of Populations

An initial AMOVA indicated low differentiation (*F*_ST_ = 0.022) with 2.17% genetic variation distributed among the 19 populations (Table 7). However, the overall *F*_ST_ differentiation was significant among the populations, based on 999 permutations (*p* = 0 < 0.001; Table 7). Even if JP_W and QD_C were removed, the overall *F*_ST_ was still significant among the remaining populations (*p* = 0 < 0.001; Table 7). Lower genetic differentiation and higher population connectivity were noted across the wild sampling sites (average *F*_ST_ = 0.015; gene flow (*Nm*) = 15.929), whereas higher genetic differentiation and lower population connectivity were noted across the hatchery sampling sites (average *F*_ST_ = 0.022; *Nm* = 10.895; Table 7).

A hierarchical AMOVA (SAMOVA) with the highest ***Φ_CT_*** was performed, and all groupings were significantly supported by permutations (0.05 ≥ *p* ≥ 0.01; Table 8). In the 19 populations analyzed, JP_W, QD_C, and others were separated (K = 3, ***Φ_CT_*** = 0.021, *p* = 0.009, variance 2.09). In the 17 populations (i.e., those other than JP_W and QD_C), the PH_W–TN_W and KS_C2–YL_W pairs were grouped (K = 15, ***Φ_CT_*** = 0.015, *p* = 0.001, variance 1.47). In the nine wild populations, when *K* = 6, the following was determined to be the optimal grouping by using the SAMOVA program [(ML_W2; ML_W3; KM_W); (PH_W; TN_W); (TP_W); (CY_W); and (ML_W1); (YL_W)]; these groups exhibited the highest intergroup variance (1.20%; Table 8). In the eight hatchery populations, when *K* = 7, [(KS_C); (PR_C1; PR_C2); (MT_C); (KS_C2); (XM_C); (KS_C3); and (KM_C)] was determined to have the highest ***Φ_CT_*** and variance (0.017 and 1.65%, respectively; Table 8).

In the STRUCTURE analysis, the best estimation of the K value (number of groups) was eight, and this corresponded to a stable representation (data not shown; Figure 2). No distinct clade pattern was noted across all populations, but the patterns for CY_W (blue), QD_C (green), and JP_W (light blue) were more clear. Despite KS (KS_C1, KS_C2, and KS_C3) and ML (ML_W1, ML_W2, and ML_W3) being from the same sampling location with three consecutive years (2015–2017), no distinct clade pattern was observed. There is a clear genetic structure change among KS and ML populations (Figure 2).

**Figure 4:**
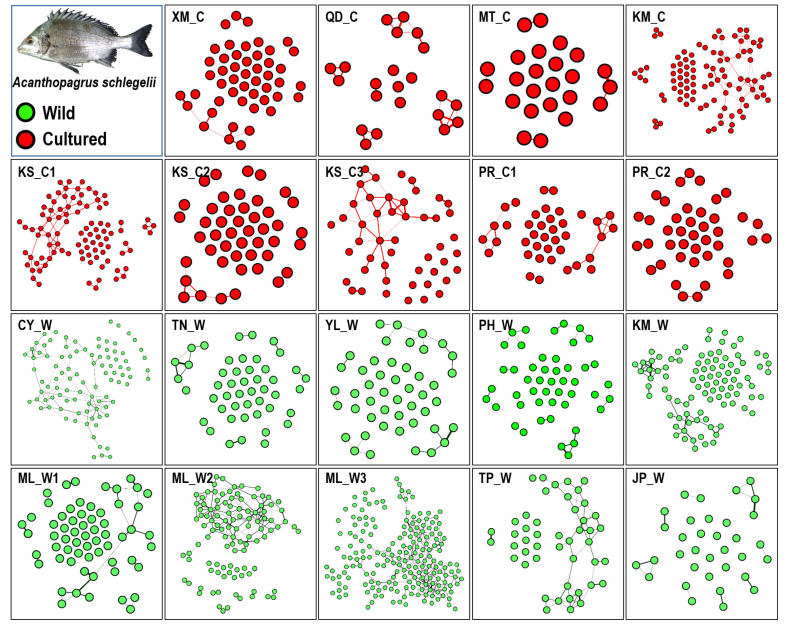
Kinship network based on pairwise relatedness within 19 populations. Wild populations: ML_W1, ML_W2, ML_W3, YL_W, PH_W, TN_W, TP_W, KM_W, CY_W, and JP_W; Cultured populations: KS_C1, KS_C2, KS_C3, PR_C1, PR_C2, KM_C, MT_C, XM_C, and QD_C. Pairwise relatedness^®^ > 0.4.

**Figure 5:**
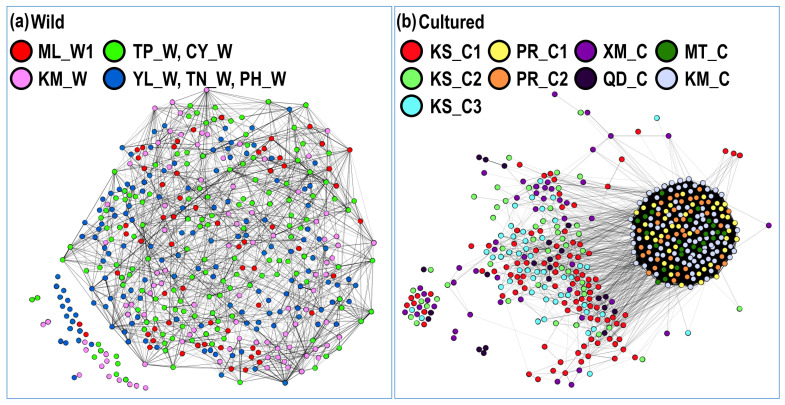
Kinship network based on pairwise relatedness: (**a**) seven wild populations and (**b**) nine cultured populations. Wild populations: ML_W1, YL_W, PH_W, TN_W, TP_W, KM_W, and CY_W; Cultured populations: KS_C1, KS_C2, KS_C3, PR_C1, PR_C2, KM_C, and MT_C. Pairwise relatedne^®^(r) > 0.4.

In the kinship network analysis based on pairwise relatedness within the 19 populations, some individuals displayed close kinship in the hatchery (KM_C, KS_C1, and KS_C3) and wild populations (CY_W, ML_W2, and ML_W3; Figure 4). However, the kinship network had a relaxed structure in seven wild populations and a concentrated structure in nine hatchery populations, indicating more inbreeding in the hatcheries (Figure 5). Parentage analysis showed that 12–49 individuals (3%–13%; 95%–80% confidence) in ML_W3 were possibly related to individuals in KS_C1 (at least a single parent; Table 9).

## 4. Discussion

### 4.1. Genetic Difference among/between Hatchery and Wild Populations

Black sea bream, *A. schlegelii,* is a crucial aquaculture species in East Asia, from Taiwan, China, and Korea to Japan. Black sea bream aquaculture began in the 1980s, and Taiwanese hatcheries are located in areas near Kaohsiung. Broodstocks are obtained from the main fishery areas, which are off the Penghu Islands and the west coast of Taiwan (Yunlin–Chiayi–Tainan–Kaohsiung). Black sea bream has been cultured for more than 30 years in Taiwan. This is the first study analyzing the genetic diversity of cultured and wild black sea bream populations in Taiwan coastal waters. Nine hatchery populations were collected, including one in northern China (as an outgroup population), QD_C (Qingdao City); one in southern China, XM_C (Xiamen city): two from Taiwan’s offshore islands, KM_C (Kinmen) and MT_C (Matsu); and five from southern Taiwan, PR_C1, PR_C2, KS_C1, KS_C2, and KS_C3 (Kaohsiung City). According to the allele number (Na), observed heterozygosity (Ho), and expected heterozygosity (He) of nine microsatellite loci, the genetic diversity of the cultured populations was slightly lower than that of the wild populations, except for some such as KS_C1 (Table 4 and Table 5). The allele frequency pattern was similar between the wild and hatchery populations (Figure 3). Genetic differences among the hatchery populations were generally larger than those among the wild populations; however, high gene flow still existed among the hatchery populations (Nm = 10.895; Table 6 and Table 7). This indicates maintenance of a high degree of genetic diversity among cultured black sea bream, and this can avoid inbreeding effects. The hatcheries used to produce juveniles for the release project during 2013–2015 (KS_C1, KS_C2, and KS_C3) imported new stock from an unknown source. Although significant changes were observed in the genetic structure, fish larvae from the unknown hatchery (PR_C1, PC_C2, and KS_C1) exhibited small genetic differences. Therefore, hatchery information is not always reliable, especially because fish larvae may come from several hatcheries simultaneously, or broodstock may change after a natural disaster. As expected, smaller genetic differences were observed among hatcheries in Taiwan than among hatcheries in Taiwan’s offshore islands, southern China, and northern China. Generally, inbreeding effects easily arise in hatchery populations due to their small effective population size, which clearly means that hatchery larvae released into the wild could reduce the genetic diversity of wild populations. For example, in one of the world’s largest marine stock early programs involving the red sea bream (*Pagrus major*) in Kagoshima Bay, Japan, the released hatchery fish clearly reduced the genetic diversity of the wild population [48]. Due to the application of a special type of stock enhancement in Taiwan (fish from private farms not from official institutes), more hatcheries (stock) could contribute to the genetic diversity, thus preventing inbreeding.

When hatchery fish are cultured, high gene flow among hatchery populations and between wild populations cannot usually be maintained. As broodstocks are not changed each year, no random mating occurs. After several generations, hatchery populations tend to show different genetic structures to wild populations [12]. Our study of the silver sea bream (*Rhabdosargus sarba*) presented two distinct clusters (hatchery and wild population clusters) [12]. As silver sea bream and black sea bream are both Sparidae fish and have a similar culture-related history in Taiwan, they are generally considered to have the same genetic structure. Unexpectedly, unlike silver sea bream, wild and hatchery black sea bream cannot be clearly separated into two clusters. The genetic structure of black sea bream did not have the same pattern among and between hatchery and wild populations. This may have had several causes; one is that the hatcheries of silver sea bream are few, located only in southern Taiwan, and maintain high communication (gene flow among silver sea bream hatcheries is high at 32.677). However, there are more hatcheries of black sea bream and they are located in different areas (i.e., southern Taiwan, offshore islands, and even China). Moreover, low but effective communication is maintained among and between populations in black sea bream hatcheries (*Nm* = 10.895). Second, black sea bream is more abundant and widely distributed in Taiwan’s coastal waters than silver sea bream. The stock population was initially established independently and from different areas and may have helped to maintain genetic diversity.

### 4.2. Dramatic Change in Genetic Structure after Fish Release

We collected 10 wild populations from the following: Japan (as an outgroup population)—JP_W (Nagasaki City); Taiwan’s offshore islands—KM_W (Kinmen) and PH_W (Penghu); northern Taiwan—ML_W1, ML_W2, ML_W3 (Miaoli City), and TP_W (Taipei City); and southern Taiwan—YL_W (Yunlin City), CY_W (Chiayi City), and TN_W (Tainan City). The fish from Taiwan’s offshore islands, northern Taiwan, and southern Taiwan were expected to have a clear genetic structure such as one population (e.g., silver sea bream) [12] or two populations (northern Taiwan vs. Penghu–southern Taiwan; e.g., rabbitfish, *Siganus fuscescens*) [49]. However, no clear pattern of genetic structure was observed among wild populations and the three wild populations (namely ML_W2, PH_W, and CY_W) that showed higher pairwise *F_ST_*. Notably, the hatchery populations KS_C1, PR_C1, and PR_C2 exhibited a lower pairwise *F_ST_* with other wild populations than with ML_W2, PH_W, and CY_W. This indicated that the hatchery populations KS_C1, PR_C1, and PR_C2 had high gene flow with the wild populations, and changes in their genetic structure were mainly caused by fish release.

Stock enhancement has been found to induce genetic structure changes in fishes such as brown trout and red sea bream [48,50]. When the genetic differences between hatchery and wild populations are considerable, the genetic structure changes greatly with a greater number of releases and longer release duration. By contrast, when the genetic difference (*F*_ST_) between KS_C1 and ML_W1 is 0, the effect on the genetic structure should be minor. However, according to the present STRUCTURE analysis, KS_C1 was different from ML_W1, and stock enhancement led to evident genetic changes over three consecutive years (ML_W1, ML_W2, and ML_W3; Figure 2). In addition, the genetic structure of sea bream from the sampling sites was inconsistent with their geographical distribution along the western coast of Taiwan. CY_W is geographically close to YL_W, TN_W, and PH_W, but the genetic structures of fish from these populations were found to be different (Figure 2). For YL_W, TN_W, and PH_W, several records of the official release from 2004 to 2015 were available. CY_W, YL_W, and TN_W were probably affected by stock enhancement and therefore exhibited no clear clusters with relative complexity in STRUCTURE analysis. For sampling sites ML_W, TP_W, and KM_W, which belonged to the northern region, several official releases were noted during 2004–2015. Long geographical distance and stock enhancement may have led to the lack of consistent and clear clusters in the STRUCTURE analysis. In addition to the large number of juveniles released over the past decade, escapes were another potential source of continued gene flow. Earth pond farming aquaculture is mainly performed in the coastal waters of southern Taiwan, and escapes happen after typhoons. Escaped farmed fish may affect natural populations and the broodstock (Holmer). One- to two-year-old silver sea bream can mature and undergo protandrous (male-to-female) sex changes later [23]. Escaped farmed fish are relatively big at 1–2 years of age and have a higher survival rate than juveniles used for stock enhancement. However, those fish were all traced to hatchery-reared stock (Figure 1). In general, fish were released over the entire western coast and outer islands of Taiwan from 2004 to 2015, resulting in complex genetic structures of wild populations that are inconsistent with their geographic distribution. Among them, no release history was found for only CY_W, which presented a simple cluster exhibiting few or no effects of stock enhancement (Figure 2).

### 4.3. Stock Enhancement of Black Sea Bream in Taiwan

Through a literature review, Araki and Schmid [51] summarized 50 years of data about the effects of hatcheries on fish and stock enhancement. They reported a clear reduction in genetic variation in hatchery populations. However, this result is completely different from the result of stock enhancement of black sea bream in Taiwan. In this study, we investigated 19 hatchery and wild populations and found that the frequency distribution of microsatellites in the hatchery and wild populations was similar, and the allele number remained at a high level (Figure 3). Additionally, we used pairwise relatedness to prepare a kinship network for evaluating the genetic relationship among each population, all hatchery populations, and all wild populations. We considered individuals with a relatedness (r) of >0.4 as related, almost excluding unrelated individuals. Weng et al. [52] used 11 microsatellites for parentage analysis in giant grouper (*Epinephelus lanceolatus*), and their relatedness value (r) of >0.25 accurately excluded unrelated individuals. In each population, we could not find obvious inbreeding groups, and further guaranteed stock enhancement should not reduce the diversity of the wild population (Figure 4). However, the kinship network indicated that the relationships among hatchery individuals were closer than those among wild individuals (Figure 5). Pairwise relatedness must be introduced to monitor stock enhancement programs and avoid unexpected inbreeding in hatcheries and large-scale programs.

Stock enhancement of black sea bream in Hiroshima Bay, Japan, is a successful example [53]. Gonzalez et al. [54] estimated that hatchery black sea bream contributed 12.5% and 13.5% to the wild population, and even as high as 58.9% in Jeong et al. [30]. In case of stock enhancement in Daya Bay, China, the contribution rate was low (approximately 1.18%), as assessed by Wang et al. [55]. Thus, stock enhancement varies widely depending on the location and method used for assessing it. In this study, the contribution rate of stock enhancement in ML_W was between 3% and 13% (Table 9). Private release (religious release) in Taiwan, another major contributor of stock, is estimated to have contributed approximately one-third of the total number of released fish. On evaluating two batches of religious release, Lee et al. [56] found that hatchery fish contributed 61% to the wild population. Regardless of the contribution rate, this study found that frequent large-scale release (official and private) in Taiwan has significantly changed the genetic structure of wild populations. Chiayi (CY_W), the main oyster production area, is a location in which no release has been recorded. Considering the influence of the oyster industry, no official stock enhancement is performed in this area, which enables investigation of the genetic structure of an area with no or few stock enhancements in Taiwan. As oyster farms are also a crucial reproductive base for black sea bream [57], the stock enhancement of black sea bream in Hiroshima Bay may have negatively affected oysters and other fishes [51,58]. Thus, not only the survival and contribution rates of released fish, but also the impacts of the release on the environment and ecology should be determined [7,59].

## 5. Conclusions

In Taiwan, official stock enhancement and private religious release of black sea bream are conducted frequently and on a large scale. Such diverse and unpredictable fish larvae prevent the decline of overall diversity. Although determining the short-term effect of stock enhancement in Taiwan is difficult, the contribution of stock enhancement to wild populations is evidenced by changes in the genetic structure and the inconsistency of such structure.

## Figures and Tables

**Table 1:** Summary of sample information. *n* = number of fish.

0	*n*	Year	Sampling Location	Fish Types	Source Information
Cultured populations					
KS_C1	94	2015	Kaohsiung, Taiwan	bloodstock	For release project during 2013–2015
KS_C2	46	2016	-	-	Import new stock from an unknown source
KS_C3	48	2017	-	-	Import new stock from an unknown source
PR_C1	39	2015	unknown hatchery	juveniles	Private (religious) release
PR_C2	41	2015	-	-	-
KM_C	96	2015	Kinmen, Taiwan	subadult/adult	Farm fish from offshore islands of Taiwan
MT_C	24	2015	Matsu, Taiwan	-	Farm fish from offshore islands of Taiwan
XM_C	48	2015	Xiamen, China	-	Farm fish from southern China
non-native					
QD_C	22	2015	Qingdao, China	-	Farm fish from northern China for comparison
Total	458				
Wild populations					
ML_W1	62	2015	Miaoli, Taiwan	subadult/adult	Release project during 2013–2015
ML_W2	106	2016	-	-	-
ML_W3	192	2017	-	-	-
YL_W	47	2015	Yunlin, Taiwan	-	Release history during 2004–2015
PH_W	48	2015	Penghu, Taiwan	-	-
TN_W	47	2015	Tainan, Taiwan	-	-
TP_W	47	2015	Taipei, Taiwan	-	-
KM_W	94	2015	Kinmen, Taiwan	-	-
CY_W	96	2015	Chiayi, Taiwan	-	No release history during 2004–2015
non-native					
JP_W	34	2015	Nagasaki, Japan	-	Wild population from Japan for comparison
Total	773				

**Table 2:** Nine selected microsatellite loci of black sea bream.

Locus	Primer Sequences (5′–3′)	Repeat Motif	Ta (°C)	Size Range (bp)	Accession No.	Reference
AS144	F: CGACGTGATGGGTTATTCTTAGAC R: GCCATTCCACAGATTTCTTTCTC	(AC)n	60	96–128	GU121415	Kim et al., 2010 [27]
AS194	F: GATCCTGTCCAGTTGCCCAGTA R: TCCACAGCTGAAACACGACTACAT	(AC)n	60	122–192	GU121416	
AS324	F: CCCAAAAACTACGTAATGCACCTT R: GCCGGATGAAGATTCTGCTC	(GT)n	60	168–238	GU121417	
AS392	F: AACCTGACCAGCCTGGCTCTTC R: ACCTCCTCTGATGCTTTTGTGTGC	(AC)nAT(AC)n	60	124–188	GU121420	
CL011	F: CCATCGCTTGACACTAGCAC R: GCCACACTTGAGCCTTTCTC	(GATA)nGATG (GATA)n	60	212–256	FJ554545	Reid et al., 2012 [28]
SaI10	F: TCACGGGGGACCAAGACTG R: CTCACACTGCCTAATTAGCACAGA	(GT)n	60	173–211	AY322107	Liu et al., 2007 [29]
SaI19	F: ATTCTTCACAGGCCCAACACAAA R: GAAAACACCGGCCCAGTACGA	(GT)n	60	232–278	AY322111	
ACS-4	F: TTTACACACCGGGAGCTCAA R: GTAAAGATCCATGGAGGTGC	(GT)n	60	76–112	AB095009	Jeong et al., 2007 [30]
AC229	F: TGTCCGTTCTGCTTTGCTC R: TGCGGTAGTGCCTTCTCTG	(TG)n	60	297–327	GU166144	Yang et al., 2014 [31]

**Table 3:** Number of alleles (*Na*) and effective alleles (*Ne*) at nine microsatellite loci in 19 black sea bream populations.

Pop (*n*)		AS144	AS194	AS324	AS392	CL011	SaI10	SaI19	ACS-4	AC229	Total
All hatchery in Taiwan (388)	*Na*	12	11	29	14	11	13	20	15	14	139
	*Ne*	2.420	3.165	15.209	3.222	6.514	1.792	5.853	5.114	4.227	Mean 5.280
All hatchery in this study (458)	*Na*	14	13	29	14	11	13	20	15	15	144
	*Ne*	2.420	3.231	16.036	3.174	6.441	1.777	5.903	5.119	4.148	Mean 5.361
All wild in Taiwan (739)	*Na*	16	17	27	15	10	13	19	17	15	149
	*Ne*	2.174	3.484	14.317	3.054	5.508	1.812	5.845	5.719	4.122	Mean 5.115
All wild in this study (773)	*Na*	16	18	29	15	11	13	19	17	15	153
	*Ne*	2.190	3.473	14.663	3.023	5.520	1.854	5.713	5.730	4.227	Mean 5.155
All populations (1231)	*Na*	17	20	31	18	12	16	21	18	17	170
	*Ne*	2.275	3.391	16.094	3.101	5.890	1.826	5.796	5.548	4.213	Mean 5.348

**Table 4:** Summary statistics for genetic variation at nine microsatellite loci in nine cultured black sea bream populations.

Pop (*n*)		AS144	AS194	AS324	AS392	CL011	SaI10	SaI19	ACS-4	AC229	Average
KS_C1 (94)	*Na*	6	6	20	8	10	8	15	9	11	10.3
	*Ne*	2.074	3.010	12.264	3.048	6.550	2.115	6.100	4.982	3.671	4.868
	*H*_o_	0.468	0.734	0.926	0.606	0.915	0.532	0.862	0.840	0.617	0.722
	*H*_e_	0.518	0.668	0.918	0.672	0.847	0.527	0.836	0.799	0.728	0.724
	*F*_IS_	0.096 ^NS^	−0.099 ^NS^	−0.008 ***	0.098 **	−0.080 ^NS^	−0.009 ***	−0.031 ***	−0.051 ***	0.152 ***	0.008
KS_C2 (46)	*Na*	9	8	20	6	10	5	13	8	8	9.7
	*Ne*	2.332	3.338	13.478	3.132	6.003	1.716	5.658	3.992	4.240	4.877
	*H*_o_	0.543	0.761	0.891	0.630	0.935	0.435	0.739	0.804	0.761	0.722
	*H*_e_	0.571	0.700	0.926	0.681	0.833	0.417	0.823	0.750	0.764	0.718
	*F*_IS_	0.048 ***	−0.086 ^NS^	0.037 ^NS^	0.074 **	−0.122 ^NS^	−0.042 ^NS^	0.102 ^NS^	−0.073 ^NS^	0.004 ^NS^	−0.006
KS_C3 (48)	*Na*	7	6	19	6	7	4	10	6	9	8.2
	*Ne*	1.983	2.703	12.659	3.388	4.174	1.475	3.882	4.067	5.525	4.428
	*H*_o_	0.583	0.729	0.917	0.667	0.771	0.354	0.771	0.688	0.854	0.704
	*H*_e_	0.496	0.630	0.921	0.705	0.760	0.322	0.742	0.754	0.819	0.683
	*F*_IS_	−0.177 ^NS^	−0.157 ^NS^	0.005 *	0.054 ^NS^	−0.014 ^NS^	−0.100 ^NS^	−0.038 ^NS^	0.088 ***	−0.043 ^NS^	−0.042
PR_C1 (39)	*Na*	5	7	19	8	8	5	9	9	9	8.8
	*Ne*	2.222	3.045	10.864	2.477	5.707	1.783	5.012	5.750	4.527	4.599
	*H*_o_	0.564	0.692	0.949	0.641	0.718	0.308	0.795	0.667	0.897	0.692
	*H*_e_	0.550	0.672	0.908	0.596	0.825	0.439	0.800	0.826	0.779	0.711
	*F*_IS_	−0.026 ^NS^	−0.031 ^NS^	−0.045 *	−0.075 ***	0.130 *	0.299 ***	0.007 ***	0.193 ***	−0.152 ^NS^	0.033
PR_C2 (41)	*Na*	11	9	19	9	7	7	12	7	9	10.0
	*Ne*	2.957	3.928	11.207	2.478	6.180	2.079	6.380	5.003	4.197	4.934
	*H*_o_	0.610	0.732	0.805	0.463	0.634	0.293	0.780	0.780	0.805	0.656
	*H*_e_	0.662	0.745	0.911	0.596	0.838	0.519	0.843	0.800	0.762	0.742
	*F*_IS_	0.079 ***	0.018 ***	0.116 ***	0.223 ***	0.243 ***	0.436 ***	0.074 ^NS^	0.025 ^NS^	−0.057 ***	0.129
KM_C (96)	*Na*	7	4	16	9	7	4	15	7	9	8.7
	*Ne*	3.143	3.098	9.974	2.805	6.002	1.387	5.635	4.266	3.488	4.422
	*H*_o_	0.677	0.667	0.740	0.729	0.781	0.240	0.802	0.802	0.760	0.689
	*H*_e_	0.682	0.677	0.900	0.644	0.833	0.279	0.823	0.766	0.713	0.702
	*F*_IS_	0.007 ^NS^	0.016 ^NS^	0.178 ***	−0.133 ^NS^	0.063 ^NS^	0.141 ^NS^	0.025 ^NS^	−0.048 ^NS^	−0.066 ^NS^	0.020
MT_C (24)	*Na*	6	4	15	9	8	6	11	10	7	8.4
	*Ne*	1.725	2.477	8.113	4.220	5.908	2.618	4.129	6.400	3.182	4.308
	*H*_o_	0.500	0.625	0.958	0.833	0.750	0.417	0.667	0.875	0.667	0.699
	*H*_e_	0.420	0.596	0.877	0.763	0.831	0.618	0.758	0.844	0.686	0.710
	*F*_IS_	−0.190 ^NS^	−0.048 ^NS^	−0.093 ^NS^	−0.092 ***	0.097 ^NS^	0.326 **	0.120 ^NS^	−0.037 ***	0.028 ^NS^	0.012
XM_C (48)	*Na*	10	5	21	7	9	4	11	9	9	9.4
	*Ne*	1.987	3.550	12.288	2.553	5.408	1.515	5.626	4.934	2.673	4.504
	*H*_o_	0.521	0.688	0.917	0.500	0.667	0.292	0.292	0.771	0.438	0.565
	*H*_e_	0.497	0.718	0.919	0.608	0.815	0.340	0.822	0.797	0.626	0.683
	*F*_IS_	−0.048 ***	0.043 ^NS^	0.002 ^NS^	0.178 ***	0.182 ^NS^	0.142 ^NS^	0.645 ***	0.033 ^NS^	0.301 ***	0.164
QD_C (22)	*Na*	5	7	11	4	6	4	5	6	7	6.1
	*Ne*	2.960	2.898	8.566	2.310	5.661	2.127	4.155	4.102	4.137	4.102
	*H*_o_	0.409	0.364	0.864	0.727	0.773	0.727	0.773	0.318	1.000	0.662
	*H*_e_	0.662	0.655	0.883	0.567	0.823	0.530	0.759	0.756	0.758	0.711
	*F*_IS_	0.382 ***	0.445 ***	0.022 ***	−0.282 ^NS^	0.061 ***	−0.372 ^NS^	−0.018 *	0.579 ***	−0.319 *	0.055

*n* = number of samples, *Na* = allele number, *Ne* = allele richness, *He* = expected heterozygosity, *Ho* = observed heterozygosity, *F_IS_* = fixation index. ^NS^ = not significant, * *p* < 0.05, ** *p* < 0.01, *** *p* < 0.001 in Hardy–Weinberg equilibrium test.

**Table 5:** Summary statistics for genetic variation at nine microsatellite loci in 10 wild black sea bream populations.

Pop (*n*)		AS144	AS194	AS324	AS392	CL011	SaI10	SaI19	ACS-4	AC229	Average
ML_W1 (62)	*Na*	7	6	19	7	9	5	12	7	10	9.1
	*Ne*	2.644	2.887	12.835	2.733	5.142	1.883	5.539	4.754	4.288	4.745
	*H*_o_	0.597	0.710	0.919	0.758	0.710	0.468	0.726	0.742	0.823	0.717
	*H*_e_	0.622	0.654	0.922	0.634	0.806	0.469	0.819	0.790	0.767	0.720
	*F*_IS_	0.040 ^NS^	−0.086 ^NS^	0.003 ***	−0.195 ^NS^	0.119 ^NS^	0.003 ^NS^	0.114 ***	0.060 ^NS^	−0.073 ^NS^	−0.002
ML_W2 (106)	*Na*	9	10	17	9	9	5	15	8	12	10.444
	*Ne*	2.254	3.012	7.531	3.205	4.858	1.462	4.640	5.247	5.368	4.2
	*H*_o_	0.566	0.736	0.877	0.689	0.755	0.330	0.792	0.774	0.689	0.690
	*H*_e_	0.556	0.668	0.867	0.688	0.794	0.316	0.784	0.809	0.814	0.700
	*F*_IS_	−0.017 ^NS^	−0.102 ^NS^	−0.012 ^NS^	−0.001 ***	0.050 ^NS^	−0.045 ^NS^	−0.010 ^NS^	0.044 ^NS^	0.154 ***	0.007
ML_W3 (192)	*Na*	10	10	21	10	9	7	14	8	13	11.333
	*Ne*	2.356	3.706	10.602	2.927	5.338	1.883	5.296	6.164	3.932	4.7
	*H*_o_	0.521	0.839	0.594	0.547	0.672	0.396	0.745	0.641	0.646	0.622
	*H*_e_	0.576	0.730	0.906	0.658	0.813	0.469	0.811	0.838	0.746	0.727
	*F*_IS_	0.095 ***	−0.148 ***	0.344 ***	0.169 ***	0.173 **	0.156 ***	0.082 ***	0.235 ***	0.134 ***	0.138
YL_W (47)	*Na*	6	6	19	7	9	6	12	10	9	9.333
	*Ne*	1.730	2.847	11.505	3.081	5.844	2.021	4.569	4.766	3.809	4.5
	*H*_o_	0.319	0.617	0.894	0.702	0.915	0.447	0.468	0.809	0.681	0.650
	*H*_e_	0.422	0.649	0.913	0.675	0.829	0.505	0.781	0.790	0.737	0.700
	*F*_IS_	0.244 *	0.049 ^NS^	0.021 *	−0.040 **	−0.104 ^NS^	0.116 ^NS^	0.401 ***	−0.023 ***	0.077 ^NS^	0.082
PH_W (48)	*Na*	8	6	21	6	8	6	9	6	10	8.889
	*Ne*	2.102	2.570	13.921	1.848	5.870	2.525	5.183	4.535	5.020	4.8
	*H*_o_	0.458	0.583	0.896	0.333	0.833	0.563	0.792	0.688	0.813	0.662
	*H*_e_	0.524	0.611	0.928	0.459	0.830	0.604	0.807	0.780	0.801	0.705
	*F*_IS_	0.126 ***	0.045 ***	0.035 ^NS^	0.273 ***	−0.004 ^NS^	0.069 *	0.019 ^NS^	0.118 ^NS^	−0.015 ^NS^	0.074
TN_W (47)	*Na*	6	5	17	7	8	5	10	6	8	8.0
	*Ne*	2.277	2.895	11.845	2.092	5.336	1.687	4.981	4.781	3.832	4.414
	*H*_o_	0.596	0.745	0.872	0.426	0.745	0.447	0.894	0.723	0.702	0.683
	*H*_e_	0.561	0.655	0.916	0.522	0.813	0.407	0.799	0.791	0.739	0.689
	*F*_IS_	−0.062 ^NS^	−0.138 ^NS^	0.047 ***	0.185 ***	0.084 ***	−0.097 ^NS^	−0.118 ^NS^	0.085 ^NS^	0.050 ^NS^	0.004
TP_W (47)	*Na*	7	8	18	7	8	6	11	7	9	9.0
	*Ne*	1.660	4.064	11.475	2.780	5.247	1.578	5.032	5.375	3.144	4.484
	*H*_o_	0.404	0.936	0.872	0.574	0.872	0.404	0.979	0.745	0.702	0.721
	*H*_e_	0.397	0.754	0.913	0.640	0.809	0.366	0.801	0.814	0.682	0.686
	*F*_IS_	−0.017 ***	−0.242 ***	0.044 *	0.103 ***	−0.078 **	−0.104 ***	−0.221 ***	0.085 *	−0.030 ^NS^	−0.051
KM_W (94)	*Na*	10	10	20	7	8	8	16	8	11	10.9
	*Ne*	2.413	3.848	10.507	3.252	5.497	1.511	6.175	4.796	4.061	4.673
	*H*_o_	0.574	0.734	0.755	0.532	0.819	0.277	0.766	0.713	0.606	0.642
	*H*_e_	0.586	0.740	0.905	0.693	0.818	0.338	0.838	0.791	0.754	0.718
	*F*_IS_	0.019 ^NS^	0.008 ***	0.165 ***	0.232 ***	−0.001 ^NS^	0.182 ***	0.086 ***	0.099 ^NS^	0.195 ***	0.110
CY_W (96)	*Na*	7	6	19	10	9	7	13	13	10	10.4
	*Ne*	1.741	3.889	9.958	3.105	5.569	2.049	6.338	6.227	3.398	4.697
	*H*_o_	0.438	0.646	0.885	0.625	0.948	0.458	0.917	0.833	0.771	0.725
	*H*_e_	0.426	0.743	0.900	0.678	0.820	0.512	0.842	0.839	0.706	0.718
	*F*_IS_	−0.028 ^NS^	0.131 ^NS^	0.016 ^NS^	0.078 ^NS^	−0.155 ***	0.105 ***	−0.088 **	0.007 ***	−0.092 ***	−0.003
JP_W (34)	*Na*	8	7	19	5	9	4	5	6	10	8.1
	*Ne*	2.532	3.066	10.557	2.388	4.429	2.183	3.256	4.587	5.928	4.325
	*H*_o_	0.618	0.765	0.882	0.588	0.500	0.147	0.735	0.706	0.912	0.650
	*H*_e_	0.605	0.674	0.905	0.581	0.774	0.542	0.693	0.782	0.831	0.710
	*F*_IS_	−0.021 ^NS^	−0.135 ^NS^	0.025 ^NS^	−0.012 ^NS^	0.354 ***	0.729 ***	−0.061 ^NS^	0.097 **	−0.097 ^NS^	0.098

*n* = number of samples, *Na* = allele number, *Ne* = allele richness, *He* = expected heterozygosity, *Ho* = observed heterozygosity, *F_IS_* = fixation index. ^NS^ = not significant, * *p* < 0.05, ** *p* < 0.01, *** *p* < 0.001 in Hardy–Weinberg equilibrium test.

**Table 6:** Pairwise *F*_ST_ values (below the diagonal) and associated *p* values (above the diagonal) among 19 black sea bream populations collected from Taiwanese hatcheries and the wild.

	KS_C1	KS_C2	KS_C3	PR_C1	PR_C2	KM_C	MT_C	XM_C	QD_C	ML_W1	ML_W2	ML_W3	YL_W	PH_W	TN_W	TP_W	KM_W	CY_W	JP_W
KS_C1	-	0 *	0 *	0.025	0.036	0 *	0 *	0 *	0 *	0.465	0 *	0 *	0 *	0 *	0 *	0 *	0 *	0 *	0 *
KS_C2	0.023	-	0 *	0 *	0 *	0 *	0 *	0.001	0 *	0 *	0 *	0 *	0.168	0 *	0 *	0 *	0 *	0 *	0 *
KS_C3	0.018	0.023	-	0 *	0 *	0 *	0 *	0 *	0 *	0 *	0 *	0 *	0 *	0 *	0 *	0 *	0 *	0 *	0 *
PR_C1	0.005	0.025	0.027	-	0.065	0 *	0.003	0 *	0.001	0.044	0 *	0 *	0 *	0 *	0.001	0 *	0 *	0 *	0.001
PR_C2	0.005	0.028	0.024	0.006	-	0 *	0 *	0 *	0.040	0.061	0 *	0 *	0 *	0.001	0 *	0 *	0 *	0 *	0 *
KM_C	0.024	0.028	0.037	0.019	0.023	-	0 *	0 *	0 *	0 *	0 *	0 *	0 *	0 *	0 *	0 *	0 *	0 *	0 *
MT_C	0.012	0.029	0.031	0.015	0.026	0.029	-	0 *	0 *	0 *	0 *	0 *	0 *	0 *	0 *	0 *	0 *	0 *	0 *
XM_C	0.024	0.012	0.034	0.028	0.031	0.030	0.028	-	0 *	0 *	0 *	0 *	0.022	0 *	0 *	0.001	0 *	0 *	0 *
QD_C	0.022	0.045	0.037	0.022	0.012	0.043	0.047	0.052	-	0 *	0 *	0 *	0 *	0 *	0 *	0 *	0 *	0 *	0 *
ML_W1	0.000	0.026	0.012	0.005	0.005	0.024	0.021	0.031	0.022	-	0 *	0 *	0 *	0.001	0 *	0 *	0 *	0 *	0 *
ML_W2	0.023	0.031	0.017	0.022	0.025	0.023	0.027	0.032	0.049	0.018	-	0 *	0 *	0 *	0 *	0 *	0 *	0 *	0 *
ML_W3	0.015	0.023	0.024	0.012	0.016	0.019	0.019	0.022	0.041	0.013	0.007	-	0 *	0 *	0 *	0 *	0.001	0 *	0 *
YL_W	0.013	0.003	0.015	0.016	0.022	0.027	0.019	0.008	0.037	0.016	0.017	0.014	-	0 *	0 *	0 *	0 *	0 *	0 *
PH_W	0.010	0.029	0.026	0.019	0.014	0.039	0.033	0.031	0.029	0.010	0.034	0.023	0.017	-	0.040	0 *	0 *	0 *	0 *
TN_W	0.011	0.016	0.025	0.012	0.014	0.024	0.029	0.016	0.029	0.012	0.026	0.013	0.014	0.006	-	0.001	0 *	0 *	0 *
TP_W	0.017	0.023	0.019	0.019	0.020	0.027	0.021	0.012	0.048	0.020	0.022	0.012	0.017	0.024	0.011	-	0 *	0 *	0 *
KM_W	0.019	0.021	0.022	0.015	0.022	0.014	0.023	0.019	0.047	0.015	0.007	0.005	0.014	0.032	0.020	0.014	-	0 *	0 *
CY_W	0.011	0.029	0.029	0.017	0.015	0.031	0.024	0.025	0.046	0.017	0.028	0.016	0.024	0.029	0.020	0.010	0.019	-	0 *
JP_W	0.022	0.051	0.052	0.018	0.032	0.054	0.032	0.056	0.042	0.020	0.049	0.035	0.040	0.034	0.042	0.051	0.041	0.041	-

*p* values (above diagonal) and their significance after Bonferroni corrections at an alpha level of 5% (*p* = 0.05/342 = 0.0001). * *p* < 0.0001.

**Table 7:** Analysis of molecular variance (AMOVA) among different black sea bream groups.

Source	Df	Sum of Squares	Mean Squares	Variance	% Total
19 populations (All)					
Among sampling localities	18	224.668	12.482	0.071	2.17
Among individuals	1212	4139.426	3.415	0.189	5.74
Within individuals	1231	3738.000	3.037	3.037	92.09
Total	2461	8102.094		3.297	100
Average *F*_ST_ value = 0.022 (*p* = 0 < 0.001); *N_m_* = 11.291					
17 populations (All without JP_W, QD_C)					
Among sampling localities	16	190.135	11.883	0.063	1.90
Among individuals	1158	3946.553	3.408	0.184	5.59
Within individuals	1175	3573.000	3.041	3.041	92.51
Total	2349	7709.689		3.287	100
Average *F*_ST_ value = 0.019 (*p* = 0 < 0.001); *N_m_* = 12.882					
9 populations (TW wild)					
Among sampling localities	8	91.506	11.438	0.051	1.55
Among individuals	730	2508.436	3.436	0.210	6.42
Within individuals	739	2228.500	3.016	3.016	92.04
Total	1477	4828.442		3.277	100
Average *F_ST_* value = 0.015 (*p* = 0 < 0.001), *N_m_* = 15.929					
8 populations (TW cultured)					
Among sampling localities	7	78.304	11.186	0.074	2.24
Among individuals	428	1438.117	3.360	0.138	4.19
Within individuals	436	1344.500	3.084	3.084	93.56
Total	871	2860.921		3.296	100
Average *F_ST_* value = 0.022 (*p* = 0 < 0.001), *N_m_* = 10.895					

**Table 8:** Hierarchical AMOVA of 19 black sea bream populations collected from hatcheries and the wild, with analysis performed using SAMOVA.

Region Groupings		*Φ_CT_*	*p*	% Variance among Groups
19 pops (All)				
K = 2	(18pops); (JP_W)	0.021	0.051	2.05
K = 3	(17pops); (JP_W); (QD_C)	0.021	0.009	2.09
K = 4	(16pops); (JP_W); (QD_C); (PH_W)	0.015	0.003	1.47
17 pops (no JP_W, QD_C)				
K = 14	(PH_W; TN_W); (PR_C1; PR_C2); (ML_W2; ML_W3); etc…	0.012	0.001	1.24
K = 15	(PH_W; TN_W); (KS_C2; YL_W); etc…	0.015	0.001	1.47
K = 16	(PH_W; TN_W); etc…	0.013	0.044	1.33
9 pops (TW wild)				
K = 5	(ML_W2; ML_W3; KM_W); (PH_W; TN_W); (TP_W; CY_W); (ML_W1); (YL_W)	0.011	0.001	1.15
K = 6	(ML_W2; ML_W3; KM_W); (PH_W; TN_W); (TP_W); (CY_W); (ML_W1); (YL_W)	0.012	0	1.20
K = 7	(ML_W2; ML_W3; KM_W); (PH_W); (TN_W); (TP_W); (CY_W); (ML_W1); (YL_W)	0.012	0.011	1.17
8 pops (TW cultured)				
K = 4	(KS_C; PR_C1; PR_C2; MT_C); (KS_C2; XM_C); (KS_C3); (KM_C)	0.016	0	1.64
K = 6	(KS_C); (PR_C1; PR_C2); (MT_C); (KS_C2; XM_C); (KS_C3); (KM_C)	0.014	0.015	1.44
K = 7	(KS_C); (PR_C1; PR_C2); (MT_C); (KS_C2); (XM_C); (KS_C3); (KM_C)	0.017	0.017	1.65

**Table 9:** Parentage analysis of black sea bream collected from hatcheries (KS_C1) and the wild (ML_W3), with analysis performed using Cervus.

Level	Confidence	Critical LOD	Assignments		Assignments	
			Observed	%	Expected	%
Strict	95	5.01	12	3	22	6
Relaxed	80	2.50	49	13	34	9
Unassigned	-	-	335	87	350	91
Total	-	-	384	100	384	100

## Data Availability

Not applicable.
